# Protocol for a Wnt reporter assay to measure its activity in human neural stem cells derived from induced pluripotent stem cells

**DOI:** 10.1016/j.crneur.2023.100095

**Published:** 2023-06-17

**Authors:** Cristine Marie Yde Ohki, Natalie Monet Walter, Michelle Rickli, José Maria Salazar Campos, Anna Maria Werling, Christian Döring, Susanne Walitza, Edna Grünblatt

**Affiliations:** a Department of Child and Adolescent Psychiatry and Psychotherapy, Translational Molecular Psychiatry, Psychiatric University Hospital Zurich, University of Zurich, Wagistrasse 12, 8952, Schlieren, Switzerland; b Neuroscience Center Zurich, University of Zurich and the ETH Zurich, Winterthurerstrasse 11, 8057, Zurich, Switzerland; c Zurich Center for Integrative Human Physiology, University of Zurich, Winterthurerstrasse 11, 8057, Zurich, Switzerland; d Biomedicine PhD Program, University of Zurich, Winterthurerstrasse 11, 8057, Zurich, Switzerland

**Keywords:** Wnt signaling, Neuropsychiatry, Induced pluripotent stem cells, Human stem cells, Neural stem cells, Reporter assay

## Abstract

The canonical Wnt signaling is an essential pathway that regulates cellular proliferation, maturation, and differentiation during neurodevelopment and maintenance of adult tissue homeostasis. This pathway has been implicated with the pathophysiology of neuropsychiatric disorders and was associated with cognitive processes, such as learning and memory. However, the molecular investigation of the Wnt signaling in functional human neural cell lines might be challenging since brain biopsies are not possible and animal models may not represent the polygenic profile of some neurological and neurodevelopmental disorders. In this context, using induced pluripotent stem cells (iPSCs) has become a powerful tool to model disorders that affect the Central Nervous System (CNS) *in vitro*, by maintaining patients’ genetic backgrounds. In this method paper, we report the development of a virus-free Wnt reporter assay in neural stem cells (NSCs) derived from human iPSCs from two healthy individuals, by using a vector containing a reporter gene (*luc2P*) under the control of a TCF/LEF (T-cell factor/lymphoid enhancer factor) responsive element. Dose-response curve analysis from this luciferase-based method might be useful when testing the activity of the Wnt signaling pathway after agonists (e.g. Wnt3a) or antagonists (e.g. DKK1) administration, comparing activity between cases and controls in distinct disorders. Using such a reporter assay method may help to elucidate whether neurological or neurodevelopmental mental disorders show alterations in this pathway, and testing whether targeted treatment may reverse these. Therefore, our established assay aims to help researchers on the functional and molecular investigation of the Wnt pathway in patient-specific cell types comprising several neuropsychiatric disorders.

## Highlights

• Our reproducible reporter system allows the assessment of the Wnt signaling activity.
• We tested this viral-free reporter in iPSC-derived neural stem cells (NSCs).
• Preserving the genetic background of NSCs allows Wnt-activity investigation.
• This analysis is feasible in NSCs from patients with neuropsychiatric disorders.

## Graphical abstract

**Figure:**
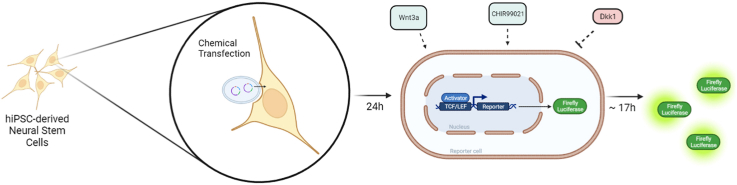

## List of abbreviations and acronyms

**Abbreviation / Acronym Definition**
μg: Microgram
μL: Microliter
μM: Micromolar
μm: Micrometer
ADHD: Attention-Deficit/Hyperactivity Disorder
APC: Adenomatous Polyposis Coli
ASD: Autism Spectrum Disorder
BASEC: Business Administration System for Ethics Committees
Bp: Base pairs
BSA: Bovine Serum Albumin
β-TrCP: Beta-Transducin repeats-containing Protein
CBCL: Child Behavior Checklist
CK1α: Casein Kinase 1α
CNS: Central Nervous System
CNV: Copy number variation
CO_2_: Carbon Dioxide
DKK1: Dickkopf Wnt signaling inhibitor-1
DMEM: Dulbecco's Modified Eagle Medium
DMSO: Dimethyl Sulfoxide
DNA: Deoxyribonucleic acid
Dvl: Dishevelled
EBs: Embryoid Bodies
EC50: Half maximal effective concentration
ESC: Embryonic Stem Cell
FOXG1: Forkhead Box G1
FZD: Frizzled
g: grams
GSK3: Glycogen Synthase Kinase 3
hESC: Human Embryonic Stem Cell
HSA: Human Serum Albumin
IC50: Half maximal inhibitory concentration
iPSC: Induced Pluripotent Stem Cell
LB: Lysogeny Broth
LDEV: Lactate dehydrogenase-elevating virus
LIN28A: Lin-28 Homolog A
LRP5/6: Lipoprotein Receptor-Related Protein 5/6
mL: Mililiter
NANOG: Nanog Homeobox Protein
NEM: Neural Expansion Media
ng: Nanogram
NSC: Neural Stem Cell
nm: nanometer
OCT4: Octamer-binding transcription factor 4
PAX-6: Paired box protein-6
PBMCs: Peripheral Blood Mononuclear Cells
PBS: Phosphate Buffered Saline
pH: Potential of Hydrogen
PSC: Pluripotent Stem Cell
QC: Quality Control
RLU: Relative Luminescence Unit
RPM: Revolutions Per Minute
RT: Room Temperature
RT-qPCR: Real Time Quantitative Polymerase Chain Reaction
SEM: Standard Error of the Mean
SeV: Sendai Virus
SOC: Super Optimal broth with Catabolite repression
SOX2: Sex determining region Y-box 2
SSEA-4: Stage-specific embryonic antigen 4
TCF/LEF: T-Cell Factor/Lymphoid Enhancer Factor
TAE: Tris-acetate-EDTA
TK: Thymidine Kinase
TRA-1-60: T cell receptoralpha locus
TRE: Transcriptional Response Element
TUJ1: Class III beta-tubulin
UV: Ultraviolet
V: Volts
Wnt: Wingless/Integrated
xg: Relative centrifugal force

## 1 Introduction

Wnt is a growth stimulatory factor, inducing cellular proliferation, polarization, and differentiation, which generally implies its relevance during developmental processes (Steinhart and Angers, 2018). Moreover, Wnt distinguishes itself from other growth factors by its exceptional ability to giving shape of expanding tissues (Nusse and Clevers, 2017). Especially during neural development, Wnt has proven to be a driving factor, from defining the anterior-posterior axis of the neural plate (Kiecker and Niehrs, 2001), regulating morphogenesis of the neural tube (van de Ven et al., 2011), modulating stem cell proliferation and differentiation in the developing and mature brain (Bengoa-Vergniory and Kypta, 2015; Gonçalves et al., 2016; Megason and McMahon, 2002), up to participating in the regulation of synaptic plasticity (Mulligan and Cheyette, 2012; Oliva et al., 2013). This outcome of Wnt-signaling is mediated by a downstream signaling cascade, activated by Wnt, which elicits changes in gene expression (Nusse and Clevers, 2017). To date, the most studied Wnt pathways are the canonical pathway, involving β-catenin as a crucial switch in gene expression, and the non-canonical pathway, independent of β-catenin (Ng et al., 2019; Zhan et al., 2017). At a molecular level, the canonical Wnt cascade can be grouped into: a) the heterodimeric receptor complex, consisting of the Frizzled receptor (FZD) and lipoprotein receptor-related protein 5/6 (LRP5/6), b) the multiprotein destruction complex, assembling axin, adenomatous polyposis coli (APC), glycogen synthase kinase 3 (GSK3), casein kinase 1α (CK1α), Beta-Transducin repeats-containing Protein (β-TrCP) and dishevelled (Dvl) proteins, and c) β-catenin (Peifer and Polakis, 2000; Taciak et al., 2018). In absence of the intercellular Wnt signal, the destruction complex binds, phosphorylates, and ubiquitinates β-catenin to be degraded by proteasomes, keeping the intracellular levels of β-catenin low (MacDonald et al., 2009). In an activated state, Wnt binds to its receptor complex, causing phosphorylation of the LRP5/6 protein. The phosphorylation evokes a destabilization of the destruction complex, resulting in an inhibition of β-catenin degradation, permitting an accumulation of free β-catenin in the cytoplasm (Okerlund and Cheyette, 2011). Consequently, free β-catenin transiently converts T-cell factor/lymphoid enhancer factor (TCF/LEF) from a transcriptional repressor to an activator, mediating gene expression (Clevers and Nusse, 2012).

The Wnt pathway is commonly active during healthy development; however, its activity also plays an important role in disease. Alterations in Wnt-signaling has been linked to central nervous system (CNS) disorders such as attention-deficit/hyperactivity disorder (ADHD), autism spectrum disorder (ASD), depression, schizophrenia, bipolar disorder, Alzheimer's disease and intellectual disability (Bem et al., 2019; Folke et al., 2019; Grünblatt et al., 2019; Hussaini et al., 2014; Kwan et al., 2016; Snijders Blok et al., 2015; Voleti and Duman, 2012; Yde Ohki et al., 2020). Available evidence presents genetic disruption in Wnt-pathway associated genes across mental disorders, affecting complex behavior, such as repetitive behavior relevant in ASD (Mulligan and Cheyette, 2017; Zhang et al., 2012), and hyperactivity/impulsivity found in ADHD (Grünblatt et al., 2018). Moreover, a variety of cancers were identified with an abnormal activation of the Wnt-pathway, encouraging cellular proliferation, migration and invasion (Taciak et al., 2018; Zhan et al., 2017). Thus, the understanding of the Wnt-pathway is essential to advance in fundamental developmental processes as well as in neurological and cancerous disease research.

Induced pluripotent stem cells (iPSCs) create a tissue relevant model for the investigation of signaling pathways in a personalized manner. iPSCs retain the same properties as embryonic stem cells (ESCs), however as iPSCs derive from patient-specific somatic cells, which are obtained non-invasively, ethical concerns as found in ESC fall away (Gurwitz, 2016). Additionally, iPSCs show their advantage in recapitulating the genetic background of patients without the need of genetic manipulation (Halevy and Urbach, 2014). This is especially crucial in mental disorders, in which a wide range of genetic variations are linked to one disease, making disease modelling with iPSCs more representative of individuals (Hunsberger et al., 2015).

Reporter gene systems are a valuable tool for investigating molecular pathway activities, such as the Wnt-pathway, by monitoring the expression of the involved promotor or transcriptional response element (TRE) sequence joined to a reporter gene, that are inserted in an expression vector (Badr and Tannous, 2011). In response to the activation of the pathway, the synthesized reporter gene transmits colorimetric, fluorescent, or luminescent signals evoked by enzymatic activities (Cheng et al., 2010). The transient delivery of the expression vector is conducted through either viral transduction or non-viral transfection. Many studies successfully performed lentiviral-mediated reporter gene expression to trace molecular pathway activities (Eggenschwiler et al., 2013; Fuerer and Nusse, 2010), as lentiviral transduction provides an efficient and stable gene expression in cell culture and *in vivo* (Shuen et al., 2015). However, a recent publication sheds awareness of potential risks that lentiviral vector exposure may carry (Schlimgen et al., 2016). The publication demonstrates studies indicating oncogenic, infectious, and other transformative alterations in the infected cells, implying a biosafety hazard. To prevent such risk, non-viral plasmid transfections provide a safe alternative of reporter gene delivery.

In the current method paper, we present an alternative transient plasmid-mediated reporter gene expression method for the observation of the Wnt activity in human iPSC-derived neural stem cells (NSCs) through chemical transfection. The induction of the cells was conducted with a vector (pGL4.49[luc2P/TCF-LEF RE/Hygro]), provided as a gift by Promega, carrying the reporter gene *luc2P* under the control of TCF/LEF transcriptional response element, eliciting a bioluminescent signal when the Wnt-pathway is activated. For signal normalization, NanoLuc Luciferase under the control of a TK (thymidine kinase) promoter was used. The successful chemical transfection of a Wnt reporter into human iPSC-derived NSCs is a novel procedure, that has not been published to date. Therefore, the performed non-viral transfection method in human iPSC-derived NSCs can be applied for detecting the Wnt signaling pathway activity across various CNS disorders.

## 2 Methods

**Fig. 1:**
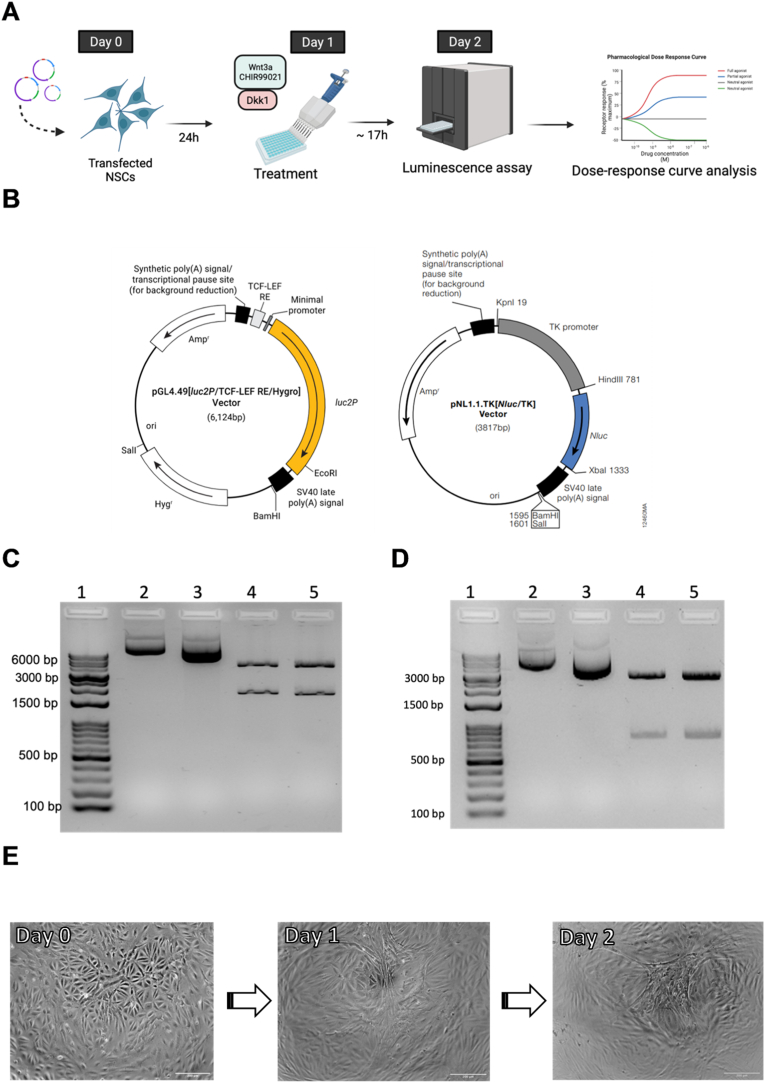
Characteristics of the vectors used in this study. (A) Experimental design of the current work containing experimental days, from the transfection of NSCs with the Wnt reporter gene until the data analysis of the luminescence assays. Image created with BioRender.com. (B) Design of the vectors pGL4.49[luc2P/TCF-LEF RE/Hygro] and pNL1.1.TK [Nluc/TK], to be cotransfected into NSCs. Figures were obtained from Promega's website. Agarose gel electrophoresis (1.5%) after overnight restriction enzyme double digestion with *Eco*RI and *Hin*dIII for pGL4.49 (C) and *Bam*HI and *Hin*dIII for the internal control vector (D). Lane 1, ladder 10 kbp; lane 2, uncut Promega plasmid; lane 3, uncut amplified plasmid; lane 4, digested Promega plasmid; lane 5, digested amplified plasmid. (E) Representative images from NSC K011 c6 in 96-wells, on days 0, 1 and 2 before transfection, treatment with agonists and luminescence assays, respectively. Scale bar: 200 μm.

An overview of the procedure is illustrated in Fig. 1A and a list of reagents is provided in Appendix 1.

### 2.1 Recruitment of individuals

The recruitment of the healthy control male children and adolescents is conducted by the Psychiatric University Hospital, Zürich, University of Zurich, as previously described (Grossmann et al., 2021; Yde Ohki et al., 2021, 2023) (Appendix 2).

All individuals and their families voluntarily consented to participate in this project, which was properly approved by the Cantonal Ethics Committee (BASEC-Nr.-2016-00101 & BASEC-Nr.-201700825) and is in accordance with the latest version of the Declaration of Helsinki.

For this study, 4 NSC lines (from two non-related healthy control male adolescents; 2 clones each) were chosen to establish and test the method (Supplementary Table 1, Supplementary Fig. 1). Additional information about the generation and culture of iPSCs and NSCs may be found in Appendix 3.

### 2.2 Plasmid amplification

In order to guarantee sufficient amount of plasmids for further transfections, the pGL4.49[luc2P/TCF-LEF RE/Hygro] Vector (kindly provided by Promega AG, E4611) and an internal control vector under the control of a TK promoter (pNL1.1.TK [Nluc/TK] Vector; Promega AG, N1501) (Fig. 1B) were separately amplified in TOP10 *E. coli* (Life Technologies, C4040-03) using heat-shock bacterial transformation. The whole 5-day process from culture to plasmid isolation and purification was performed as described in Appendix 4.

### 2.3 Passaging NSCs for further transfection

➢ **DAY -1 –**100% confluent NSCs in 6-wells were dissociated and 20′000 NSCs seeded onto each 96-well of a 96-well plate, coated with hESC-Qualified Matrigel® Matrix (Corning® Matrigel, LDEV-free, 354277), in order to be transfected on the following day.
  1) Media from the wells containing NSCs was removed.
  2) The NSCs were washed with PBS 1x, pH 7.4 (Gibco™, 10010023) (1 mL/well).
  3) StemPro™ Accutase™ (Gibco™, A1110501) (1 mL/well) was used to harvest the cells for a maximum of 3 min at 37 °C.
  4) 1 mL of PBS (Phosphate buffered saline) 1x was added to each well to inactivate the Accutase.
  5) To remove the Accutase, cells were centrifuged for 4 min at 300×*g*.
  6) The supernatant was removed and the cell pellet was resuspended in 1 mL of Neural Expansion Media (NEM).
  7) Cells were counted using Trypan Blue 0.4% (Invitrogen™, T10282), Countess™ cell counting chamber slides (Invitrogen™, C10228) and the automated cell counter Countess® II FL (ThermoFisher, ZGEXSCCOUNTESS2F).
  8) 20′000 NSCs were seeded per well of a Matrigel coated 96-well plate.
  9) Plates were incubated overnight at 37 °C under 5% CO_2_.

### 2.4 Transient chemical transfection of NSCs

➢ **DAY 0 –** To insert the reporter gene and the normalization vector into our NSCs, these cells were chemically co-transfected with the previously amplified pGL4.49[luc2P/TCF-LEF RE/Hygro] Vector and pNL1.1.TK [*Nluc*/TK] Vector by using FuGENE® HD Transfection Reagent (Promega, E2311) in a DNA:reagent ratio of 2:1, as follows:
  1) FuGENE® HD reagent stored at 4 °C was calibrated to reach room temperature (RT) and mixed by inverting. Obs.: If there were any visible pellets on the bottom of the vial, the vial was briefly warmed at 37 °C and further, cooled down to RT.
  2) To transfect the amount of 26 wells of a 96-well plate, 2 μg of pGL4.49[luc2P/TCF-LEF RE/Hygro] Vector and 20 ng of the pNL1.1.TK [*Nluc*/TK] Vector were added to a polypropylene tube, followed by 100 μL of Opti-MEM™ I Reduced Serum Medium (Gibco™, 31985070). The proportions were properly adjusted experiment-by-experiment, according to the total number of wells to be transfected.
  3) 4 μL of FuGENE® HD were added to the mixture.
  4) The solution was incubated for 15 min at RT without harsh pipetting.
  5) After incubation, the bottom of the tube was gently flipped and 4 μL of the solution was added to each 96-well. **Important:** reservoirs and multichannel pipettes were not and should not be used!
  6) Transfected plates were incubated overnight at 37 °C under 5% CO_2_.

### 2.5 Treating transfected NSCs with Wnt signaling agonists

➢ **DAY 1.1 –** On the next day, the treatment with Wnt-agonists was completed using Wnt3a (Abcam, ab81484) or CHIR-99021 (MedChemExpress, HY-10182). The Wnt3a concentrations used in this study were 0 (vehicle), 5, 10, 20, 40, 60, 80, 100, 150, 200, 400, 600 and 800 ng/mL. CHIR-99021 concentrations included: 0 (vehicle), 0.1, 0.2, 0.3, 0.6, 1.3, 1.3, 2.5, 5, 10 and 20 μM.

Stock solutions of Wnt3a at 10 μg/mL and CHIR-99021 at 1 mM were prepared: Wnt3a was resuspended in PBS containing 0.1% endotoxin-free HSA (Human Serum Albumin) (Proteintech®, HZ-3001) and stored for a longer time period at −20 °C, while CHIR-99021 was resuspended in Dimethyl Sulfoxide (DMSO; PanReac AppliChem, A3672) and stored at −80 °C. PBS and DMSO were the vehicles used for Wnt3a and CHIR-99021, respectively.

Cells were incubated overnight (for at least 17 h) at 37 °C under 5% CO_2_ conditions at a total volume of 50 μL/well.

### 2.6 Treating transfected NSCs with DKK1

➢**DAY 1.2 –** For the treatment with the Wnt antagonist DKK-1, 111 ng/mL of Wnt3a (corresponding to ca. EC26 of the analyzed cells) was added 10 min prior to DKK-1 treatment, in 40 μL/well, while additional 10 μL of DKK1 in different concentrations (0, 4, 10, 20, 30, 40 and 50 ng/mL) were added into each well. Therefore, the total volume after both treatments remained 50 μL/well. Cells were incubated overnight (for at least 17 h) at 37 °C under 5% CO_2_ conditions.

To increase its lifetime, DKK1 (Sigma-Aldrich®, SRP3258-10UG) was resuspended in water containing 1% Bovine Serum Albumin (BSA, Sigma-Aldrich®, A7030-50G) and the stock solution was stored at −20 °C. Vehicle consisted of water.

### 2.7 Luminescence assay

➢ **DAY 2 –** On the next day of overnight treatment, transfected NSCs were submitted to the luminescence assay in the dark, according to the instructions of the Nano-Glo® Dual-Luciferase® Reporter Assay System (Promega AG, N1610).
  1) ONE-Glo™ EX Reagent was added to each well (50 μL/well).
  2) The plate was incubated for 3 min at RT, at a constant agitation of 150 RPM.
  3) Cell lysates were transferred to a white clear-bottom 96 well plate (Berthold Technologies, 24910).
  4) Firefly luminescence activity was immediately measured. For our experiments, we used the luminometer function of a Mithras^2^ LB 943 Multimode Reader (Berthold Technologies) and the MikroWin 2010 (version 5.18) software. The luminometer's parameters were set up as: HiSens mode, with 0.1 s of integration time with a 60-s delay applied prior to the measurement to ensure no excitation due to light exposure before entrance to the machine is measured.
  5) Next, 50 μL of the NanoDLR™ Stop & Glo® was added to each well.
  6) A thorough pipetting up and down was performed.
  7) The plate was incubated for 10 min at RT.
  8) Nanoluc luminescence signal was immediately measured using the same settings mentioned on step 4.
  9) The raw data was exported as an Excel file for further analysis.

### 2.8 Single x double transfection

To ensure that the transfection efficiency for both plasmids were the same, we performed single transfections (either with the pGL4.49[luc2P/TCF-LEF RE/Hygro] vector or the pNL1.1.TK [*Nluc*/TK] vector) and compared the Relative Luminescence Unit (RLU) ratios with the ones obtained from double transfections. Singly and doubly transfected NSCs were then treated with three concentrations of each agonist: Wnt3a (0, 40 and 600 ng/mL) and CHIR-99021 (0, 2.5 and 10 μM). Three and two independent experiments were performed for Wnt3a and CHIR-99021, respectively.

RLU ratios from singly transfected NSCs were calculated by dividing RLU values from individual wells transfected only with the pGL4.49[luc2P/TCF-LEF RE/Hygro] vector by the mean of RLUs from wells transfected with pNL1.1.TK [*Nluc*/TK] only (within the same treatment condition).

### 2.9 Data analysis

RLU values from the Firefly Luciferase activity were normalized by the Nanoluc Luciferase signal, well by well. Normalized RLUs were cleaned using the Interquartile Range (IQR) method. For each technical replicate within a cell line, the highest RLU value was set as having Wnt activity as 100% and the other individual values were calculated relatively to it. For DKK1, the mean of RLU values from wells that were treated with Wnt3a EC26 + DKK1's vehicle were set as 26%.

From each cell line, the highest mean of RLUs (considered two technical replicates per cell line) were constrained to 100% of Wnt activity in the case of agonists. As for DKK1, the lowest mean of RLU values were constrained as bottom values. For each condition, the Wnt activity in percentages were plotted in graphs in function of the agonist/antagonist concentration. EC50 and IC50 values (defined as the half-maximal effective and inhibitory concentrations, respectively) were obtained by performing a non-linear regression for each Wnt agonist on GraphPad Prism 9.4.1. EC50 and IC50 values were calculated using GraphPad Prism 9 (version 9.4.1).

## 3 Results and discussion

In this study, a reproducible viral-free Wnt reporter assay in human iPSC-derived NSCs was developed (Fig. 1A). Non-viral methods of delivery are less immunogenic and less mutagenic, which offer a great advantage to the use of lentiviruses in reporter systems (Hardee et al., 2017; Loring et al., 2016). For this, we used the Firefly luciferase gene (*luc2P*) present in the vector pGL4.49[luc2P/TCF-LEF RE/Hygro], as a Wnt reporter under the control of a TCF-LEF responsive gene, allowing the assessment of luminescence signals after various drug treatments that can be translated into Wnt-signaling activity regulation (Fig. 1A and B).

While this method paper describes the successful transient transfection of NSCs, it should be noted that the stable transfection of iPSCs has initially been assessed. Our initial goal was to further differentiate stably transfected iPSCs (selected through a Hygromycin treatment at 70 μg/mL for one week) into NSCs, which would be submitted to treatment with Wnt agonists and luminescence assays. However, the stable transfection process might be considered more laborious, since the constant selection of clones within the cell population is necessary (Fus-Kujawa et al., 2021). The choice of using iPSCs was based on their indefinite self-renewal potential compared to those of a multipotent lineage (Liang and Zhang, 2013).

However, in our experience, the stable transfection of iPSCs through electroporation based on a protocol from Chatterjee et al. (2011) (Chatterjee et al., 2011) have yielded inconsistent results, indicating low success rate in our cells. Due to these inconsistent results of different experiments conducted with NSCs post-transfection (*i.e.*, different concentration ranges for agonists, integration time, luminescence settings), we further tested whether the plasmid was truly functionally present in the transfected iPSCs. Interestingly, when we performed luminescence assays with transfected Hygromycin-selected iPSCs treated overnight with Wnt3a, we observed that the luminescence signals were very low and inconsistent as similarly observed in the NSCs post-transfection. Supplementary Table 3 shows weak RLU values obtained from the exemplary transfected iPSC K005 z13, while Supplementary Table 4 provides the RLU values from NSCs post-transfection derived from the same cell line under different conditions following treatment with agonists.This observation could be related to a possible partial integration of the vector into the host genome, which would confer Hygromycin resistance to the cells and the formation of different subclones that might not have acquired the luciferase gene. These results have led us to pursue a new alternative that we describe in the current paper: transiently transfecting NSCs, which is a non-integrative method (e.g., not interfering with the host genome, which might be advantageous to studying disorders with genetic etiology) and, unlike required for stable transfection, does not involve the use of antibiotics for selection of successfully transfected cells (Fus-Kujawa et al., 2021).

Following, an efficient transfection method has been chosen. Although electroporation is a common delivery method for stem cells, which are usually described as more resilient to transfection, the method includes high chances of increased cell death and permanent damage (Chong et al., 2021). This led us to choose a chemical transfection using FuGENE® HD (a non-liposomal reagent), which has been previously reported in human embryonic stem cells, being the reagent with the lowest toxicity in this cell type (Suzuki et al., 2008).

To eliminate well-to-well variances (*i.e.,* transfection efficiency and variability in cell number) and obtain trustworthy results, a normalizing vector containing a Nanoluc Luciferase gene under the control of a constitutive promoter (TK) was co-transfected with our vector of interest (Fig. 1B).

Since the transient transfection is a continuous process to be repeatedly performed throughout experiments, it might be necessary to expand the amount of available material. To do so, we transformed competent *E. coli* with our plasmids, and extracted the amplified DNA after bacterial culture. However, since the use of selective antibiotics might mildly affect the stability of our plasmid following amplification (Wein et al., 2020), we performed a diagnostic enzymatic reaction by restriction enzymes. We found that the integrity of the plasmid was preserved and its original format was intact. Given that *Hin*dIII and *Eco*RI cleave the pGL4.49 vector at positions 437 bp and 2162 bp, respectively, a 6124 bp band for uncut samples and two bands for the digested ones (1724 bp and 3961 bp bands) were to be expected.

On the other hand, by cleaving the Nanoluc vector with *Hin*dIII and *Bam*HI at positions 781 and 1595 respectively, the expected products from the double digestion would have 814 bp and 3003 bp. The agarose gels further confirmed that the structure of both vectors has not been affected during the amplification process in *E. coli* (Fig. 1C and D).

Before starting a transient transfection protocol in the target cells of interest, researchers must consider some important factors. For instance, it should not only be considered how the reporter gene will be delivered, but also how transfection efficiencies may vary according to the nature of the cell lines. In this context, the cell type to be targeted (Kim et al., 2015), size (Wang et al., 2021), cell cycle stage (Brunner et al., 2000) and differentiation stage (Stein and Schindler, 2011) could be determinant to a successful transfection. Due to that, the different methodological origins of NSC generation could reflect in a wide range of NSC phenotypes (Galiakberova and Dashinimaev, 2020), which may result in different experimental outcomes than those described in our paper. For our case, the process does not seem to morphologically affect the transfected and treated NSCs, as representatively depicted in Fig. 1E.

Although it is not possible to determine whether each cell was transfected with both of our vectors, the molecular biology community well accepts that a correlated packaging of both plasmids occurs when a transfection reagent is added to the mixture (Gam et al., 2019). Nevertheless, to prove that the transfection was efficient for our vectors, we compared RLU ratios when the NSC line K015 c1 was submitted to single and double transfections. Non-significant differences between single and double transfected wells suggest that the transfection process in both conditions are similar (Supplementary Fig. 2).

**Fig. 2:**
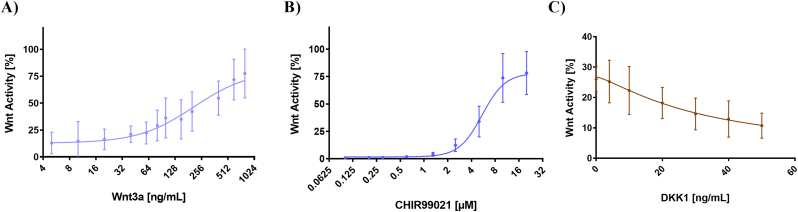
Dose-curve response after treatment of NSCs with different concentrations of the Wnt-agonists Wnt3a (A) and CHIR-99021 (B), as well as the antagonist DKK1 (C). In the latter, the activity is normalized to 26%, as the cells were treated with Wnt3a′s EC26 (111 ng/mL) prior to the addition of DKK1. The mean and standard deviation (SD) of the four cell lines were taken together to generate a non-linear regression curve (details on non-linear regression statistics can be found in Supplementary Table 6). In the graphs, the highest Wnt activity (100%) for Wnt3a and CHIR-99021 was set as the maximum mean of individual percentages obtained when the four cell lines are plotted together. Conversely, for DKK1, 26% was determined as the mean of the individual percentages from wells treated with 0 ng/mL DKK1 (containing Wnt3a′s EC26).

The success of our transient transfection process was functionally observed by the RLU values (Supplementary Table 5) and the ascending dose-curve responses following NSC treatment with the Wnt-agonists, CHIR-99021 and Wnt3a in all four cell lines (two clones from two different individuals) (Fig. 2A and B, Supplementary Figs. 3 and 4). An additional approach to verify the functionality was assessed by inhibiting Wnt-signals through the treatment of the cells with DKK1, a Wnt-antagonist (Fig. 2C, Supplementary Fig. 5). Individual EC50 and IC50 values of each NSC line can be found in Table 1, while their regression can be reviewed in Supplementary Figures 2, 3 and 4.

**Table 1:** Mean and standard deviation (SD) of the resulted EC50 and IC50 values from each cell line.

Cell line	EC50		IC50
	Wnt3a (ng/mL)	CHIR-99021 (μM)	DKK1 (ng/mL)
K011 c6	90.01 ± 20.6	4.74 ± 0.59	25.04 ± 4.45
K011 c10	239.25 ± 18.6	6.05 ± 0.56	11.21 ± 3.05
K015 c1	218.70 ± 45.1	5.10 ± 0.05	18.47 ± 6.65
K015 c9	362.45 ± 88.1	7.59 ± 2.8	11.22 ± 3.01

Wnt3a-conditioned media has previously been used in reporter assays as an alternative to the use of purified Wnt3a in human NSCs (Zhao et al., 2012). However, Wnt3a-conditioned media consists of an unknown environment containing undefined factors that might promote differentiation of stem cells (Tüysüz et al., 2017). On the other hand, the high hydrophobicity of purified Wnt3a may be an issue for serum-free media or serum-free diluents. In our case, the presence of HSA (a maximum negligible concentration of 0.008%) was used as a stabilizer that guaranteed the promotion of Wnt activity, as recommended by the manufacturer.

When our NSC lines were analyzed together as one single group, the EC50 values for Wnt3a and CHIR-99021 were 196.4 ng/mL and 5.272 μM, respectively. Our results show that our NSCs are responsive to different Wnt signaling agonists acting on distinct points of the cascade: Wnt3a, an extracellular ligand that binds to LRP5/6 and Frizzled (MacDonald and He, 2012); and CHIR-99021, antagonizes the downstream GSK3β as reviewed by Law and Zheng (2022) (Law and Zheng, 2022).

We also observed that increasing concentrations of the Wnt-inhibitor, DKK1, gradually reduced Wntactivity in our NSCs (Fig. 2C, Supplementary Fig. 5). The IC50 value obtained by the non-linear regression curve was 19.31 ng/mL. Statistical details of the non-linear regression after analyzing the four cell lines together can be found in Supplementary Table 6.

In neuropsychiatry, the Wnt signaling has been previously investigated in human NSCs from idiopathic autism (Marchetto et al., 2017) and Pitt-Hopkins Syndrome patients (Papes et al., 2022), which might suggest the association of some of their phenotypes with an imbalance in Wnt activity. Not only neurodevelopmental disorders, but also neurodegenerative diseases (such as Alzheimer's and Parkinson's Disease) have been linked to the Wnt dysregulation as reviewed by Gao and colleagues (Gao et al., 2021). Therefore, elucidating the functionality of the Wnt signaling in human neural cell lines, while investigating CNS disorders at the molecular level is crucial for a deeper understanding of their pathophysiology. Furthermore, since the Wnt pathway coordinates essential cellular processes during embryonic development, neurodevelopment, neurogenesis, and sustainability of cells, understanding its activity alterations may explain the fundamental etiology of certain disorders (Mulligan and Cheyette, 2012). Subsequently, these results might facilitate the search for potential novel therapeutic targets involving the Wnt pathway.

## Funding

We would like to acknowledge the Fonds für wissenschaftliche Zwecke im Interesse der Heilung von Geisteskrankheiten (Nr. 8702) and the 10.13039/100012107Waterloo Foundation (reference number 2462/4548) for funding.

## CRediT authorship contribution statement

**Cristine Marie Yde Ohki:** Methodology, Validation, Visualization, Formal analysis, Investigation, Writing – original draft. **Natalie Monet Walter:** Validation, Visualization, Formal analysis, Investigation, Writing – original draft. **Michelle Rickli:** Validation, Visualization, Formal analysis, Investigation, Writing – original draft. **José Maria Salazar Campos:** Investigation. **Anna Maria Werling:** Resources, Writing – review & editing. **Christian Döring:** Resources, Writing – review & editing. **Susanne Walitza:** Resources, Funding acquisition, Writing – review & editing. **Edna Grünblatt:** Conceptualization, Resources, Supervision, Project administration, Funding acquisition, Writing – review & editing.

## Declaration of competing interest

The authors declare the following financial interests/personal relationships which may be considered as potential competing interests: Edna Grünblatt reports a relationship with Waterloo Foundation that includes: funding grant. Edna Grünblatt reports a relationship with MEDICE Arzneimittel Pütter GmbH & Co. KG that includes: funding grant.

Susanne Walitza reports a relationship with Swiss National Science Foundation that includes: funding grants. Susanne Walitza reports a relationship with European Commission Seventh Framework Programme for Research that includes: funding grants. Susanne Walitza reports a relationship with Gesamtstrategie Hochspezialisierte Medizin of the Canton of Zurich that includes: funding grants. Susanne Walitza reports a relationship with Federal Institute for Drugs and Medical Devices that includes: funding grants. Susanne Walitza reports a relationship with Zürcher Impulsprogramm zur nachhaltigen Entwicklung der Psychiatrie that includes: funding grants. Susanne Walitza reports a relationship with Hartmann Müller Stiftung that includes: funding grants. Susanne Walitza reports a relationship with Olga Mayenfisch Foundation that includes: funding grants. Susanne Walitza reports a relationship with Gertrud Thalmann Fonds of the UPK Basel that includes: funding grants. Susanne Walitza reports a relationship with Vontobel Foundation that includes: funding grants. Susanne Walitza reports a relationship with Uniscientia Foundation that includes: funding grants. Susanne Walitza reports a relationship with Erika Schwarz Foundation that includes: funding grants. Susanne Walitza reports a relationship with Gesundheitsförderung Schweiz that includes: funding grants. Susanne Walitza reports a relationship with Georg Thieme Verlag KG that includes: consulting or advisory. Susanne Walitza reports a relationship with Hogrefe Publishing Group that includes: consulting or advisory. Susanne Walitza reports a relationship with W Kohlhammer GmbH that includes: consulting or advisory. Susanne Walitza reports a relationship with Springer Nature Deutschland GmbH Heidelberg that includes: consulting or advisory. Susanne Walitza reports a relationship with Beltz Publishing Group that includes: consulting or advisory.

All other authors have no known competing financial interests or personal relationships that could have appeared to influence the work reported in this paper.

## Data Availability

Data will be made available on request.
