# Supplementary material for: Protocol for a Wnt reporter assay to measure its activity in human neural stem cells derived from induced pluripotent stem cells
Source: Curr Res Neurobiol. 2023 Jun 17;5:100095. doi: 10.1016/j.crneur.2023.100095 (PMC10329100; doi:10.1016/j.crneur.2023.100095)
Supplement: Multimedia component 2 [file mmc2.pdf]

Current Research in Neurobiology
Part of the CO+RE suite of journals
LEARN MORE
OPEN ACCESS

Author Q&A: Protocol for a Wnt reporter assay to measure its activity in human neural stem cells derived from induced pluripotent stem cells (CRNEUR-D-23-00021R1)

Could you tell us a little bit about the basis for your study and how it helps to advance the scientific field?

In our methodological paper, we aimed to develop a protocol that could functionally assess in induced pluripotent stem cells (iPSCs)-derived Neural Stem Cells (NSCs) the Wnt signaling activity, an essential cellular pathway for neurodevelopmental processes that has been implicated with multiple neuropsychiatric disorders.

To do so in a personalized manner, we have used human iPSC-derived NSCs as models, in which each line preserves the genetic background of their respective donors, which is an advantageous foundation of personalized medicine. However, NSCs might be considered a “hard-to-transfect” cell type in a halting manner. Given this context, we have adjusted and optimized a fast and feasible reporter assay in human NSCs. Therefore, this novel approach would help researchers effectively determine the individual Wnt activity of each line in an easy manner, which might be a crucial factor in understanding disorder-specific cellular and molecular mechanisms but also study substances effects on this pathway.

What were the scientific or other challenges that you faced and how did you overcome them?

Before choosing to transiently transfect our NSCs with our vector of interest, we have tried to generate stably transfected iPSC lines and subsequently submit them to NSC generation, given that iPSCs usually have higher recovery potential than NSCs. However, this attempt was proven unsuccessful since low luminescence signals were emitted from the luminescence assays after the treatment with increasing concentrations of Wnt agonists. Since the transfected iPSCs and generated NSCs remained alive after weeks of Hygromycin selection (the gene present in the transfected plasmid), our initial thought was that the Wnt reporter vector was probably being expressed in our cells. Therefore, we tested different conditions, such as the range concentration of agonists and reading parameters.

However, after extensive trials and brainstorming sessions, we have found out that the iPSCs also presented extremely low luminescence signal values, even before the neural induction process, which might indicate the integration of the antibiotic resistance into the cells without the plasmid. This led us to change our approach to a transient transfection, since the accidental formation of subclones could be possible in the stable transfection protocol.

Readers might be interested in aspects that go beyond the scientific paper published. For instance, is there something about your perseverance individually or the team that you think made it possible to succeed with your research? Did you benefit from having a diversity of perspectives as part of the research either from your team or beyond?

Teamwork was definitely one key factor for the development of this paper in all of its aspects. High levels of resilience and hard work are inherent characteristics of our team.

Additionally, interdisciplinary discussions also proved to be fruitful. On some occasions, insightful advice from scientists who research in other biomedical areas strongly were undoubtedly necessary and contributed to move toward our final goal and overcome scientific drawbacks we faced along the way. This was specifically the case of a Physiology professor at the University of Zurich, who

suggested us that partial formation of subclones could have happened in our transfected iPSCs and led to inconsistent results.

Are there any insights that you would like to share with other investigators or those thinking about whether to dedicate their careers to studying the brain? How do you think your work could also encourage more individuals from under-represented backgrounds to get involved in neuroscience?
Neuroscience research is a wonderful path that should not be limited to only one specific type of research group. Great ideas might get off the drawing board by adapting protocols and adjusting into more economic and sustainable (and still safe) practices. This is specifically valid for research groups that are limited to low budget and/or to investment cuts in science.
As members of a group mostly composed by women, we also believe that gender by itself should not be considered as a determinant factor when it comes to producing high-quality science, with great creativity, motivation and teamwork.

Did you take advantage of some of our journal options (like double blind review) and how did you find the transparent review option?
No, we did not. Our article has been submitted to a Single-Anonymized peer review.

We would very much like to get your and your colleagues thoughts on our journal innovations (Editorial Introduction to the journal here) by way of our survey if you have the time: https://www.surveymonkey.co.uk/r/5LHWTML. Please feel free to circulate to other colleagues.

Brief author bio and pictures, if you choose:

Please add a brief biography (~25 words) of at least the lead authors with a picture if you so choose.

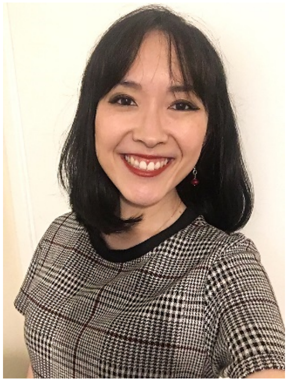

Cristine Marie Yde Ohki

Cristine is a PhD student from the Biomedicine PhD graduate program at the University of Zurich, investigating the effects of Methylphenidate in ADHD-derived neural cell lines and its involvement with the Wnt signaling pathway.

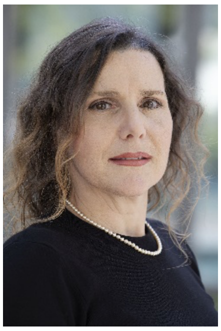

Edna Grünblatt

Edna Grünblatt is the head of the Translational Molecular Psychiatry research at the Department of Child and Adolescent Psychiatry and Psychotherapy, Psychiatric University Hospital Zurich (PUK), Chair of the ECNP iPSC platform for Neuropsychiatry Network and a Professor at the University of Zurich, Switzerland. Her main research focus on neurodevelopmental mental disorders, including ADHD, ASD, psychosis (to schizophrenia) and early onset OCD. She has established research both at the pre-clinical as well as at the basic molecular neuroscience, integrating both approaches into a translational research. The goal of her research is to elucidate the etiopathology of the disorders discovering biomarkers for early diagnosis and precision personalized medicine, predicting treatment response and outcomes.

Congratulations on your scientific achievement and thank you for helping CRNEUR to continue to innovate.

The CRNEUR Editorial Team
