# Supplementary material for: Protocol for a Wnt reporter assay to measure its activity in human neural stem cells derived from induced pluripotent stem cells
Source: Curr Res Neurobiol. 2023 Jun 17;5:100095. doi: 10.1016/j.crneur.2023.100095 (PMC10329100; doi:10.1016/j.crneur.2023.100095)
Supplement: Multimedia component 3 [file mmc3.pdf]

Protocol for a Wnt reporter assay to measure its activity in human neural stem cells derived from induced pluripotent stem cells

Cristine Marie **Yde Ohki**[1,4], Natalie Monet **Walter**[1], Michelle **Rickli**[1], José Maria **Salazar Campos**[1], Anna Maria **Werling**[1], Christian **Döring**[1], Susanne **Walitza**[1,2,3], Edna **Grünblatt**[1,2,3]

[1] Department of Child and Adolescent Psychiatry and Psychotherapy, Translational Molecular Psychiatry, Psychiatric University Hospital Zurich, University of Zurich, Wagistrasse 12, 8952 Schlieren, Switzerland

[2] Neuroscience Center Zurich, University of Zurich and the ETH Zurich, Winterthurerstrasse 11, 8057 Zurich, Switzerland

[3] Zurich Center for Integrative Human Physiology, University of Zurich, Winterthurerstrasse 11, 8057 Zurich, Switzerland

[4] Biomedicine PhD Program, University of Zurich, Winterthurerstrasse 11, 8057 Zurich, Switzerland

*** Correspondence:**
Edna Grünblatt
edna.gruenblatt@kjpd.uzh.ch

Supplementary Material

Appendix 1: List of reagents and specific material

- Culture of NSCs
  - Pluripotent Stem Cell (PSC) Neural Induction Medium (ThermoFisher, A1647801)
  - Corning® Matrigel® hESC-Qualified Matrix, LDEV-free (Corning®, 354277)
  - Dulbecco's Modified Eagle Medium /Nutrient Mixture F-12 (DMEM/F12) (Gibco™, 11320033)
  - Advanced DMEM/F-12 (Gibco™, 12634028)
  - StemPro™ Accutase™ (Gibco™, A1110501)
  - Phosphate Buffered Saline (PBS) 1x, pH 7.4 (Gibco™, 10010023)
  - 96-well plates (Sarstedt, 83.3924)
  - Countess™ cell counting chamber slides (Invitrogen™, C10228)
  - Countess® II FL (ThermoFisher, ZGEXSCCOUNTESS2F)
  - Trypan Blue Stain 0.4% (Invitrogen™, T10282)
- Plasmid amplification and extraction
  - pGL4.49[luc2P/TCF-LEF RE/Hygro] Vector (Promega AG, E4611)
  - pNL1.1.TK [Nluc/TK] Vector (Promega AG, N1501)
  - TOP10 *E. coli* (Life Technologies, C4040-03)
  - Lysogeny Broth (LB) Agar (Roth AG, X965.2)
  - Super Optimal broth with Catabolite repression (SOC) medium (Invitrogen, 15544-034)
  - Ampicillin (Sigma-Aldrich, A9393-5G)
  - LB Broth medium (Roth AG, X964.2)
  - ZymoPURE™ II Plasmid Midi Prep kit (Zymo Research, D4200)
- Enzymatic digestion of amplified vectors
  - 10X NEBuffer 3.1 (New England BioLabs, B7203)
  - 10X NEBuffer 2.1 (New England BioLabs, B7202S)
  - EcoRI (10 units) (New England BioLabs, R0101S)
  - BamHI (20 units) (New England BioLabs, R0136S)
  - HindIII (20 units) (New England BioLabs, R0104)
- Detection of digested vectors for integrity investigation
  - 10,000 bp-ladder (CSL-MDNA-BP, from Cleaver Scientific)
  - Gel Loading Dye, Purple (6x) (New England Biolabs, B7024S)
  - HDGreen™ Plus DNA Stain (Intas, ISII-HDGreen Plus)
  - SeaKEM agarose (Lonza, 182126)
  - Tris-acetate-EDTA (TAE) 1x (PanReac AppliChem, A4227,1000)
- Transient transfection of NSCs
  - FuGENE® HD Transfection Reagent (Promega, E2311)
  - Opti-MEM™ I (Improved Minimum Essential Medium) Reduced Serum Medium (Gibco™, 31985070)
- Treatment of transfected NSCs
  - Wnt3a (Abcam, ab81484)
  - CHIR-99021 (MedChemExpress, HY-10182)
  - Dickkopf Wnt Signaling Inhibitor-1 (DKK1) (Sigma-Aldrich®, SRP3258-10UG)
  - Endotoxin-free Human Serum Albumin (HSA) (Proteintech®, HZ-3001)
  - Bovine Serum Albumin (BSA, Sigma-Aldrich®, A7030-50G)
  - Dimethyl Sulfoxide (DMSO; PanReac AppliChem, A3672)

- Luminescence assays
    - Nano-Glo® Dual-Luciferase® Reporter Assay System (Promega AG, N1610)
    - White clear-bottom plates (Berthold Technologies, 24910)

Appendix 2: Additional information about subject recruitment

The subjects were investigated with the following clinical assessment:

- Wechsler Intelligence test for Children (Kaufman et al. 2006)
- Kiddie Schedule for Affective Disorders and Schizophrenia (which consists of semi-structured interview with the individual and one of his/her parents; (Kaufman et al. 1997);
- Family Interview for Genetic Studies (Preisig et al. 1999);
- Conner's- 3 rating scales for ADHD diagnosis (questionnaire for subjects, parents and teachers; (Lidzba et al. 2013);
- Child Behaviour Checklist (CBCL; questionnaire for parents; (Achenbach 1991).

For healthy controls in the current paper, the inclusion criteria was Intelligence Quotient (IQ) higher than 75 in the Wechsler Intelligence test, as well as T-scores lower than 60 in Conners-3 rating scales (hyperactivity, inattentiveness, and impulsivity ADHD scales). Additionally, these individuals must not be diagnosed or present any positive indication of any other neuropsychiatric condition (according to Kiddie Schedule for Affective Disorders and Schizophrenia and the Family Interview for Genetic Studies).

Appendix 3: Generation and culture of iPSC-derived NSCs

Plucked hair-derived keratinocytes or peripheral blood mononuclear cells (PBMCs) from two healthy individuals (K011 and K015, respectively) were reprogrammed into iPSCs via Sendai virus transduction (Yde Ohki et al. 2021; Yde Ohki et al. 2023). Quality control (QC) techniques included Copy number variation (CNV) analysis for detection of genomic aberrations introduced by the reprogramming, mycoplasma testing, gene and protein expression analysis of pluripotency markers in iPSCs (through real time quantitative polymerase chain reaction (RT-qPCR) and immunocytochemistry, respectively), generation of Embryoid Bodies (EBs) and gene expression analysis of markers of the three germ layers (ecto-, endo- and meso-derm), and detection of Sendai virus traces by RT-qPCR, as described in Yde Ohki et al. (Yde Ohki et al. 2021; Yde Ohki et al. 2023).

Quality control of the iPSC lines generated from the healthy individuals K011 & K015 showed gene expression of the pluripotency markers (*NANOG*, *OCT4*, *LIN28A* and *SOX2*), negligible detection of Sendai virus (SeV) in culture, absence of mycoplasma in the supernatant of iPSCs and positive protein expression of SSEA4, OCT4, TRA-1-60 and SOX2. Moreover, CNV analysis showed no genetic aberrations that could compromise our studies (Yde Ohki et al. 2021; Yde Ohki et al. 2023).

After performing the QC steps with iPSCs, these cells were submitted to a Neural Induction protocol for 7 days (PSC Neural Induction Medium, ThermoFisher, A1647801) and expanded until they reached at least passage 5, for QC assessing morphology, proteins and gene expression markers expected in NSCs (Supplementary Figure 1).

NSCs were cultured in Matrigel coating (Corning® Matrigel® hESC-Qualified Matrix, LDEV-free 354277), diluted 1:100 in DMEM F-12 (Gibco™, 11320033) and in Neural Expansion Media (NEM), from ThermoFisher Scientific (PSC Neural Induction Medium, A1647801), with which the cells were refreshed every other day or every two days, depending on confluence and consumption. This media is composed by Advanced DMEM/F-12 (Gibco™, 12634028) and Neurobasal Medium in a 50:50 ratio plus Neural Induction Supplement, according to the manufacturer protocol. The latter is provided as part of the PSC Neural Induction Medium (Gibco™, A1647801), as well as Neurobasal medium.

Appendix 4: Protocol for plasmid amplification and subsequent diagnostic restriction enzyme digestion

1. Plasmid amplification

- **DAY 1 – LB Agar preparation, bacterial transformation and seeding**

LB Agar preparation

1. At least 2 hours prior to the transformation, 35g of LB Agar (Roth AG, X965.2) were diluted in one liter of autoclaved distilled water, using a magnetic stirrer for a few seconds at room temperature (RT).
2. Agar was autoclaved for about 2 hours. Meanwhile, bacterial transformation reaction was prepared.

Bacterial transformation

1. Under sterile conditions, one aliquot (30-50 µL) of TOP10 *E. coli* was thawed on ice and slowly transferred into 2-mL tubes, avoiding bubble formation.
2. 1 µL of pGL4.49[luc2P/TCF-LEF RE/Hygro] Vector or pNL1.1.TK [Nluc/TK] Vector was carefully added to the bacteria, while gently swirling the pipette inside the solution.
3. The solution was incubated on ice for 30 minutes.
4. Heat-shock for 30 seconds at 42°C was performed and the tube was returned to ice for 2 minutes.
5. 250 µL of SOC medium (Invitrogen, 15544-034) was added and the tube was incubated for 1 hour at 37°C in a shaking incubator (250 RPM).

Seeding the transformed bacteria

1. Still under sterile conditions, the autoclaved liquid agar bottle was placed into a water bath at 50ºC to avoid polymerization.
2. Once the liquid reached 50ºC, 100 µg/mL Ampicillin (Sigma-Aldrich, A9393-5G) was added to the agar.
3. The solution was poured into Petri dishes, and they were left at RT for polymerization.
4. Once polymerized, the plates were turned upside down until use.
5. 100 µL of transformed *E. coli* was seeded on a Petri dish containing LB agar and Ampicillin 100 µg/mL. Varying seeding concentrations may be performed due to possible variations in bacterial transformation and growth rates (e.g., in another plate, spread 200 µL and in a third one, spread 300 µL).
6. The agar plates were then incubated overnight at 37°C for selection of transformed *E. coli*.

- **DAY 2 – Plates were removed from the incubator and placed in the fridge for processing on day 3**

➢ DAY 3 – Selecting and growing transformed bacteria

LB Broth preparation

1. LB Broth medium (Roth AG, X964.2) was prepared by weighing 25g per liter of autoclaved distilled water.
2. Medium was autoclaved for about 2 hours.
3. Once it is done and reaches 50ºC, add the Ampicillin 100 µg/mL.

Selection of colonies

1. First, a big, isolated colony was scratched with a sterile pipette tip and inoculated in 5 mL LB + Ampicillin solution in a 50 mL falcon, partially closed to allow oxygen entrance and bacterial growth.
2. In another 50 mL falcon tube, properly labelled, a small, isolated colony was inoculated under the same conditions.
3. Both tubes were submitted to a 6-hour pre-culture at 37°C.
4. After 6 hours, inoculate the bacteria solution from the big colony was diluted 1:1000 in 150 mL of LB Broth (in other words, 150 µL of it) in an Erlenmeyer.
5. In another Erlenmeyer, 250 µL of the small-colony-solution were transferred to 150 mL of LB Broth. **Important**: these dilutions might vary according to the proliferative capacity of the bacteria.
    a. There might be some competition between very proliferative bacteria, and it causes the DNA isolated from big colonies to decrease. Therefore, it is recommended to isolate a small colony in parallel.
6. The two Erlenmeyers were covered with an aluminum foil and placed in a shaker for 12-18 hours at 37°C.

➢ DAY 4 – The bacterial solution was centrifuged at 6,800 xg for 3 minutes at room temperature, and the pellet cryopreserved at -20°C until processing on day 5.

➢ DAY 5 – Performing a Midi Prep DNA extraction

The ZymoPURE™ II Plasmid Midi Prep kit (Zymo Research, D4200) was used to obtain endotoxin-free plasmids, according to the manufacturer's instructions.

- After DNA extraction, the plasmid concentration was measured spectrophotometrically (230, 260 and 280 nm) using NanoDrop (ThermoFisher)
- Samples were stored at -20ºC.

2. Diagnostic restriction enzyme digestion

To verify the integrity of the amplified plasmids, we performed a diagnostic digestion with restriction enzymes: HindIII and EcoRI were chosen to digest the amplified pGL4.49[luc2P/TCF-LEF RE/Hygro] Vector, whereas HindIII and BamHI were used to digest the amplified pNL1.1.TK [*Nluc*/TK] Vector. More details can be found on Supplementary Table 2 and figure 1B.

The reaction mix was incubated overnight at 37ºC, and the enzymes inactivated for 20 minutes at 80ºC on the following day. Negative controls included both amplified and Promega plasmids without any restriction enzymes.

To identify the size of digested products, an agarose gel (1.5%) was prepared in a 200 mL Erlenmeyer (1.5 g of SeaKEM agarose (Lonza, 182126) in 100 mL of Tris-acetate-EDTA (TAE) Buffer 1x (PanReac AppliChem, A4227,1000), by using a microwave to heat up the mixture from 30 to 30 seconds until complete dissolution of agarose particles. It is important to use a tall container since the hot agarose can boil over. **Attention:** stir the hot agarose carefully, to avoid spills and burns. The solution was stirred for around 10-15 minutes, until it reached a temperature around 50ºC. Following, 5 µL of HDGreen^{TM} Plus DNA Stain (Intas, ISII-HDGreen Plus) was added to the agarose for DNA detection. The solution was poured onto an agarose gel tray with a 15-pin comb and polymerized for around 40 minutes at RT.

Samples were loaded into the gel using a Gel Loading Dye, Purple (6x) (New England Biolabs, B7024S) diluted to 1x with 0.1668 µg of digested plasmid. For uncut samples, the same amount was applied. A 10,000 bp-ladder (CSL-MDNA-BP, from Cleaver Scientific) was used to distinguish band sizes and the gel was run for around 30 minutes at 110V. The Bio-Rad ChemiDoc^{TM} XRS + System and the Image Lab^{TM} software (version 6.0.0) were used to visualize bands in the gel under the ultraviolet (UV) light followed by acquisition of the pictures.

A)

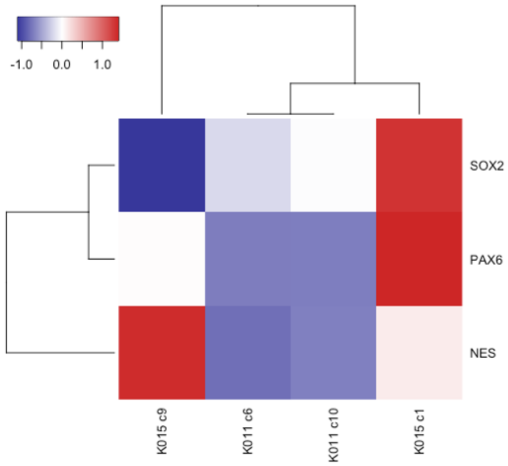

B)

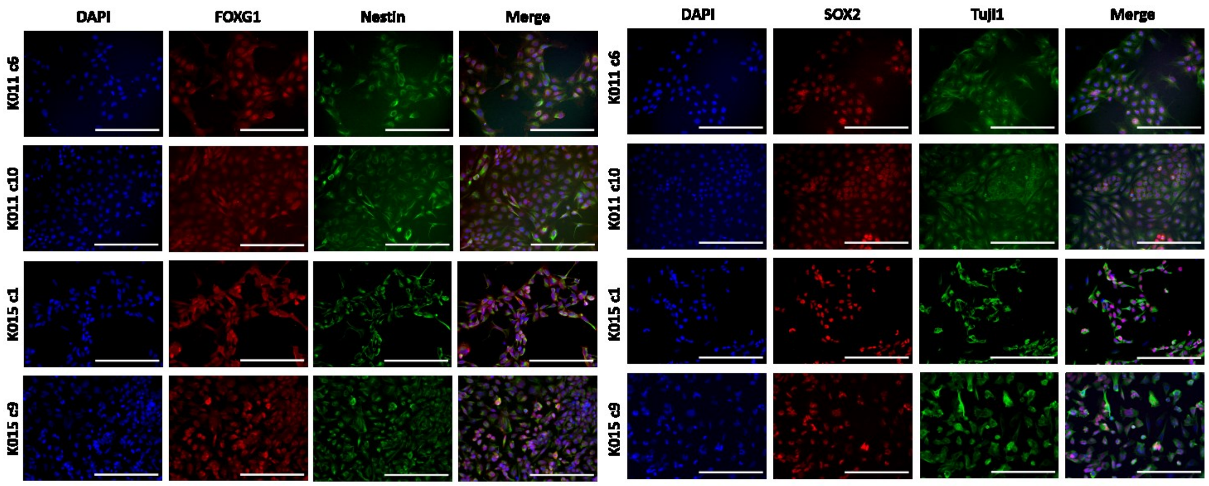

Supplementary Figure 1. Quality control of the NSCs K011 and K015. (A) Heatmap showing gene expression of the NSC markers *SOX2*, *PAX6* and *NESTIN (NES)*. (B) Immunofluorescent images show positive protein expression of TUJ1, NESTIN, SOX2 and FOXG1. Scale bar: 200 µm.

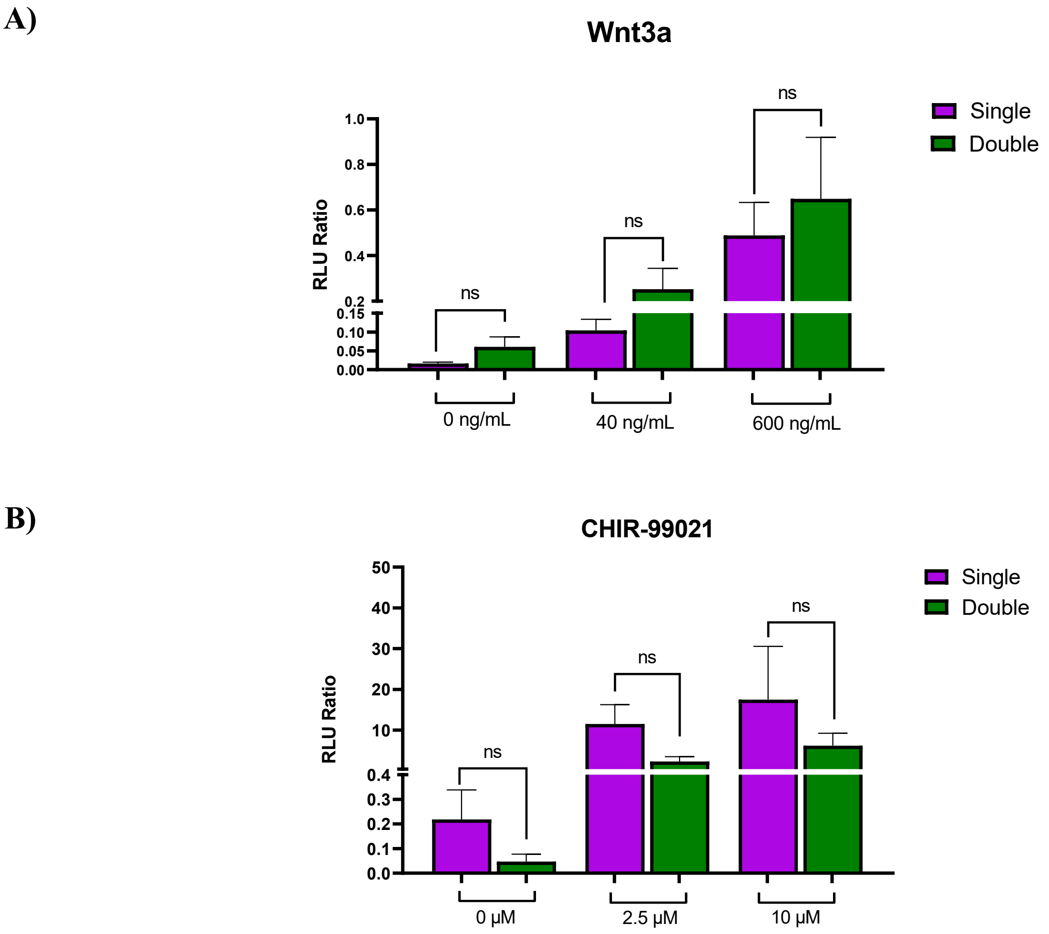

Supplementary Figure 2. Comparisons between RLU ratios from single (purple) and double transfections (green) of NSC K015 c1. The graphs represent three different concentrations of the two agonists, Wnt3a (A) and CHIR-99021 (B), depicted individually. Mann-Whitney tests were applied to compare RLU ratios from single and double transfections (ns). Mean ± SEM are represented in the graphs.

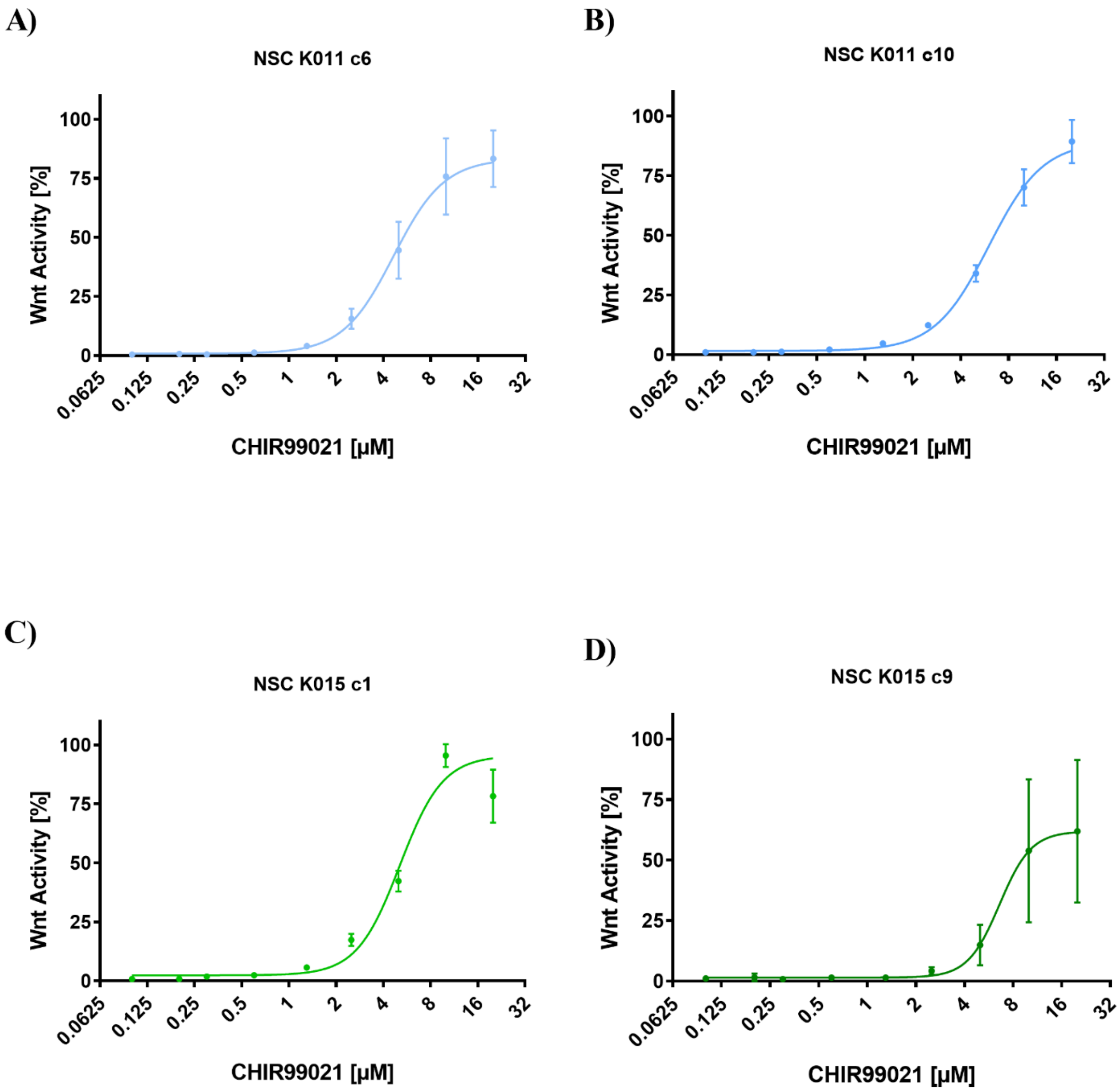

Supplementary Figure 3. Individual dose-response curves after treatment with different concentrations of CHIR-99021 in NSCs K011 c6 (A), K011 c10 (B), K015 c1 (C) and K015 c9 (D).

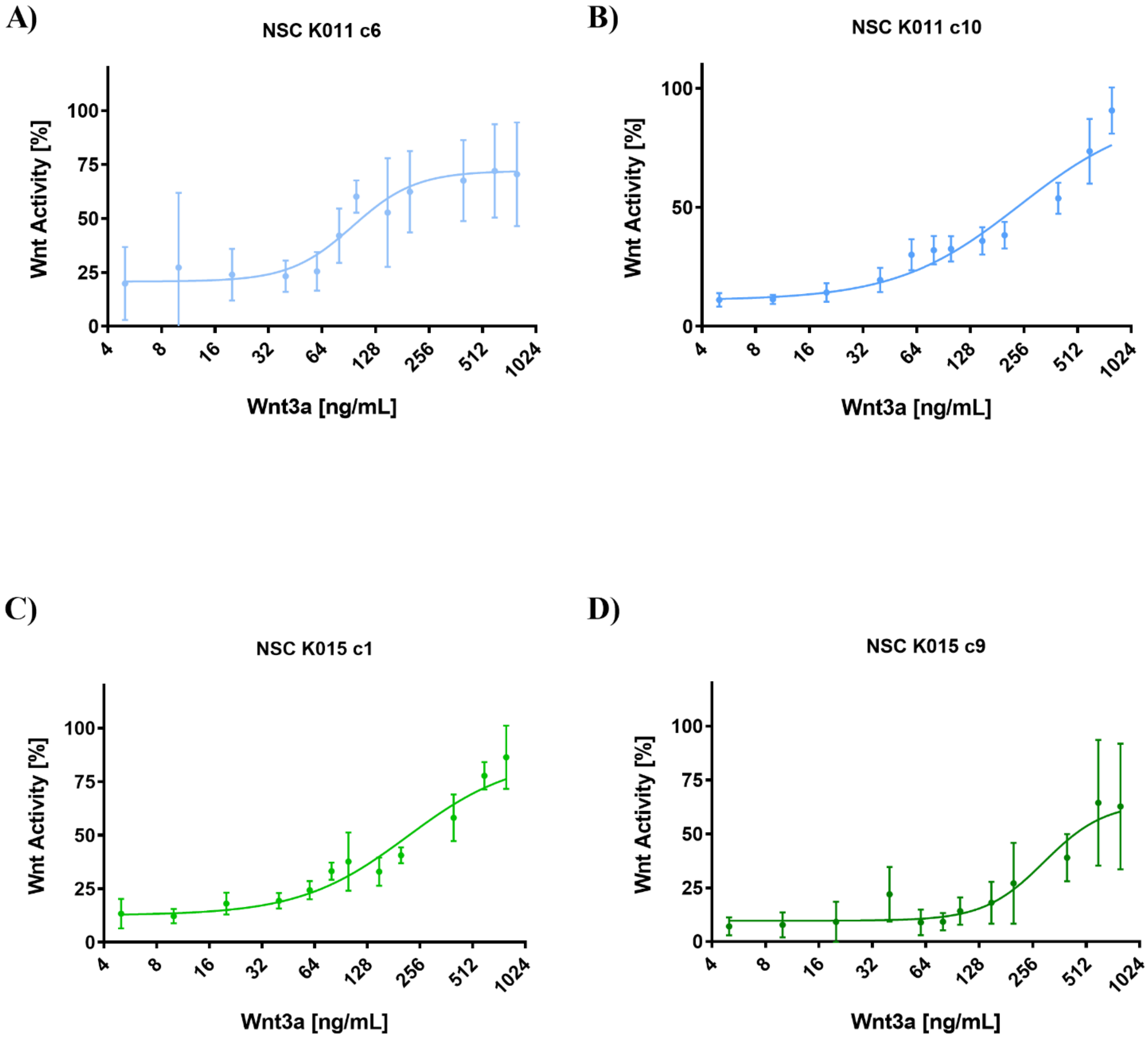

Supplementary Figure 4. Individual dose-response curves after treatment with different concentrations of Wnt3a in NSCs K011 c6 (A), K011 c10 (B), K015 c1 (C) and K015 c9 (D).

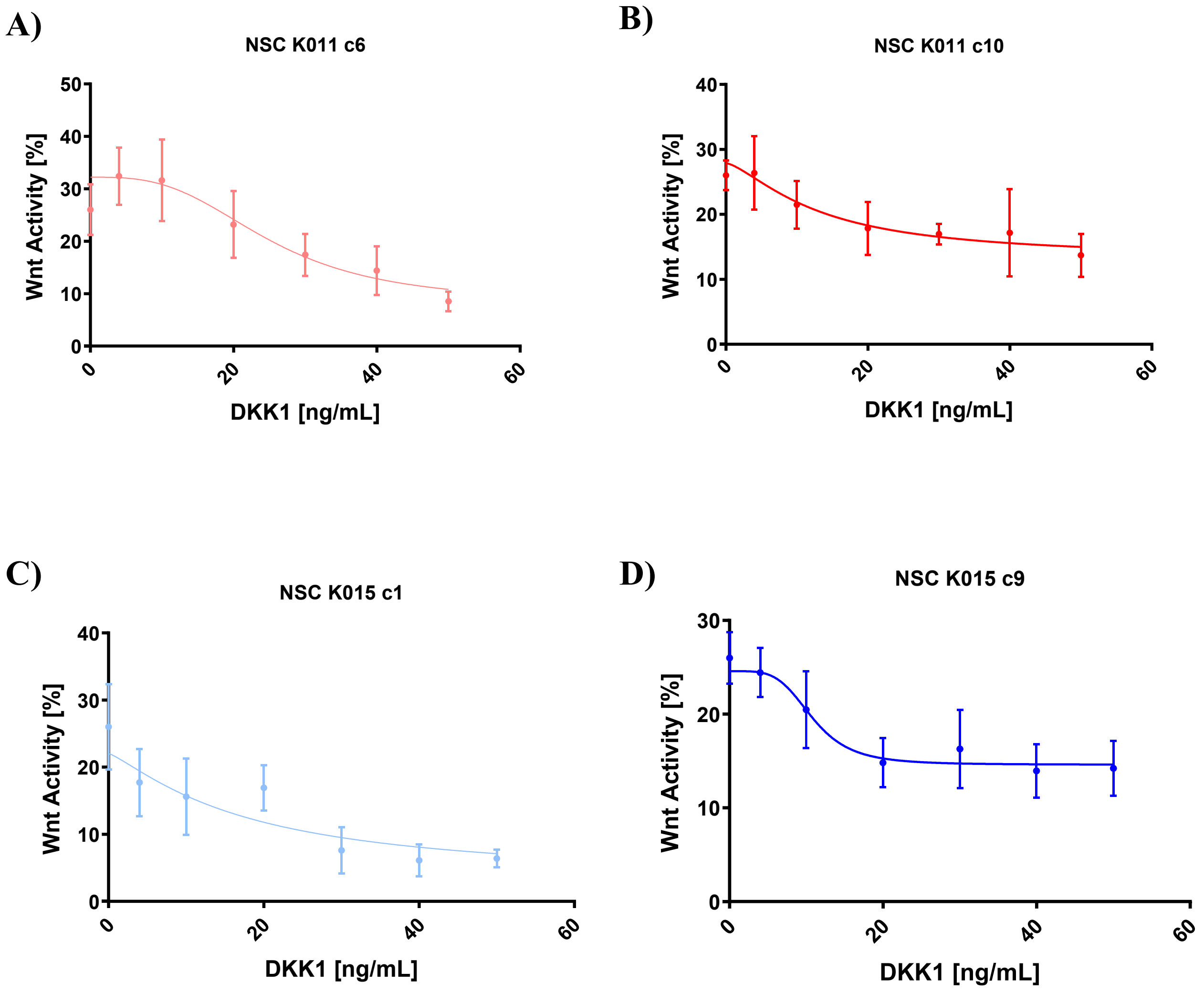

Supplementary Figure 5. Individual dose-response curves after treatment with different concentrations of the Wnt antagonist DKK1 in NSCs K011 c6 (A), K011 c10 (B), K015 c1 (C) and K015 c9 (D), after pre-treatment with Wnt3a's EC26.

Supplementary Table 1. Demographic data from the lines included in this study**.**

Cell line	Clone	Diagnosis	Age (years old)	Gender	IQ
K011	c6	Healthy control	16	Male	124
	c10				
K015	c1	Healthy control	13	Male	127
	c9				

Supplementary Table 2. Enzymatic digestion of the vectors used in this methodology.

COMPONENT	**pGL4.49[luc2P/TCF-LEF RE/Hygro] Vector**	**pNL1.1.TK [*Nluc*/TK] Vector**
**DNA**	1 µg	1 µg
**10X NEBuffer**	5 µL (1X) of 10X NEBuffer 3.1 (New England BioLabs, B7203)	5 µL (1X) of 10X NEBuffer 2.1 (New England BioLabs, B7202S)
**Enzyme 1**	1 µL of EcoRI (10 units) (New England BioLabs, R0101S)	1 µL of BamHI (20 units) (New England BioLabs, R0136S)
**Enzyme 2**	1 µL of HindIII (20 units) (New England BioLabs, R0104)	1 µL of HindIII (20 units) (New England BioLabs, R0104)
**Nuclease-free water**	to 50 µL	to 50 µL

Supplementary Table 3. Relative Luminescence Units (RLU) under different conditions of exemplary stably transfected iPSC K005 z13, after overnight treatment with Wnt3a.

	**Average RLU**			**Integration time: 1 second**
**Wnt3a (ng/mL)**	10'000 iPSCs per 96-well	20'000 iPSCs per 96-well	25'000 iPSCs per 96-well	

0	40	40	35	
5	40	60	65	
10	45	40	50	
20	46.67	50	50	
40	50	60	40	
80	50	50	55	
100	50	30	50	
150	50	66.67	60	
200	60	40	46.67	
	**Average RLU**			**Integration time: 10 seconds**
**Wnt3a (ng/mL)**	10'000 iPSCs per 96-well	20'000 iPSCs per 96-well	25'000 iPSCs per 96-well	
0	433	428	453	
5	428	453	448	
10	433	457.5	452.5	
20	398	418	408	
40	431.33	443	373	
80	418	418	398	
100	507	378	477.5	
150	474	537	383	
200	398	517	428	

Supplementary Table 4. Relative Luminescence Units (RLU) under different conditions of exemplary NSCs post-transfection K005 z13, after overnight treatment with Wnt3a.

	**Average RLU**			
**Wnt3a (ng/mL)**	20'000 NSCs per 96-well	25'000 NSCs per 96-well	30'000 NSCs per 96-well	**Integration time: 10 seconds**
0	606.67	411.33	361.33	
5	434.67	384.67	404.67	
10	404.67	364.67	388.00	
20	391.33	384.67	414.33	
40	361.33	391.33	428	
80	391.33	384.67	431.33	
100	371.33	378	421.33	
150	401.33	431.33	404.67	
200	474.33	421.33	394.67	

Supplementary Table 5. Maximum and minimum Relative Luminescence Units (RLU) for each cell line and treatment.

Plasmid	Treatment / Cell line	Wnt3a		CHIR-99021		DKK1	
		Max	Min	Max	Min	Max	Min
pGL4.49[luc2P/TCF-LEF RE/Hygro]	K011 c6	16381	10	3431	20	1124	70
	K011 c10	77201	2656	325980	1571	65972	1611
	K015 c1	37238	905	136509	746	1333	30
	K015 c9	497	10	875	10	2695	845
pNL1.1.TK [*Nluc*/TK]	K011 c6	30186	229	1154	129	2437	308
	K011 c10	136489	56125	106551	10583	495022	158330
	K015 c1	7231	1392	36552	766	2427	1273
	K015 c9	1054	149	816	40	9797	5729

Supplementary Table 6. Statistical information from non-regression curve analysis.

Treatment	Analyzed cell lines	HillSlope (Best-fit values)	R Squared	Sum of Squares	Sy.x	Degrees of Freedom	Number of x values	Number of Y values analyzed
Wnt3a	K011 c6/c10 + K015 c1/c9 combined	1.441	0.6386	77493	15.86	308	312	311
CHIR-99021		3.360	0.8837	26428	10.63	234	240	237
DKK1		-2.482	0.4927	5450	5.747	165	168	168
Wnt3a	K011 c6 only	2.443	0.5415	25988	18.61	75	78	78
CHIR-99021		2.682	0.9523	2916	7.216	56	60	59
DKK1		-3.114	0.6712	1264	5.693	39	42	42

Wnt3a	K011 c10 only	1.257	0.8838	5647	8.678	75	78	78
CHIR-99021		2.554	0.9839	938.6	4.058	57	60	60
DKK1		-1.516	0.5386	678.6	4.171	39	42	42
Wnt3a	K015 c1 only	1.409	0.8724	6240	9.121	75	78	78
CHIR-99021		3.175	0.9529	3108	7.450	56	60	59
DKK1		-1.604	0.5941	1065	5.225	39	42	42
Wnt3a	K015 c9 only	2.571	0.6479	15309	14.38	74	78	77
CHIR-99021		4.429	0.7646	9111	12.76	56	60	59
DKK1		-2.615	0.6964	388.5	3.156	39	42	42

Supplementary References

Achenbach, Thomas M. (1991): Manual for the child behavior checklist. Burlington, Vt.: Dept. of Psychiatry.

Kaufman, Alan S.; Flanagan, Dawn P.; Alfonso, Vincent C.; Mascolo, Jennifer T. (2006): Test Review: Wechsler Intelligence Scale for Children, Fourth Edition (WISC-IV). In *Journal of Psychoeducational Assessment* 24 (3), pp. 278–295. DOI: 10.1177/0734282906288389.

Kaufman, J.; Birmaher, B.; Brent, D.; Rao, U.; Flynn, C.; Moreci, P. et al. (1997): Schedule for Affective Disorders and Schizophrenia for School-Age Children-Present and Lifetime Version (K-SADS-PL): initial reliability and validity data. In *Journal of the American Academy of Child and Adolescent Psychiatry* 36 (7), pp. 980–988. DOI: 10.1097/00004583-199707000-00021.

Lidzba, Karen; Christiansen, Hanna; Drechsler, Renate (2013): Conners-3. Conners Skalen zu Aufmerksamkeit und Verhalten - 3. Deutschsprachige Adaptation der Conners 3rd EditionTM (Conners 3TM) von C. Keith Conners. Bern: Verlag Hans Huber.

Preisig, M.; Fenton, B. T.; Matthey, M. L.; Berney, A.; Ferrero, F. (1999): Diagnostic interview for genetic studies (DIGS): inter-rater and test-retest reliability of the French version. In *European archives of psychiatry and clinical neuroscience* 249 (4), pp. 174–179. DOI: 10.1007/s004060050084.

Yde Ohki, Cristine Marie; Grossmann, Leoni; Döring, Christian; Hoffmann, Per; Herms, Stefan; Werling, Anna Maria et al. (2021): Generation of integration-free induced pluripotent stem cells from healthy individuals. In *Stem cell research* 53, p. 102269. DOI: 10.1016/j.scr.2021.102269.

Yde Ohki, Cristine Marie; Walter, Natalie Monet; Rickli, Michelle; van Puyenbroeck, Pakita; Döring, Christian; Hoffmann, Per et al. (2023): Generation of induced pluripotent stem cells from two ADHD patients and two healthy controls. In *Stem cell research* 69, p. 103084. DOI: 10.1016/j.scr.2023.103084.
