# Supplementary material for: Protocol for a Wnt reporter assay to measure its activity in human neural stem cells derived from induced pluripotent stem cells
Source: Curr Res Neurobiol. 2023 Jun 17;5:100095. doi: 10.1016/j.crneur.2023.100095 (PMC10329100; doi:10.1016/j.crneur.2023.100095)
Supplement: Multimedia component 1 [file mmc1.pdf]

# Peer Review Overview

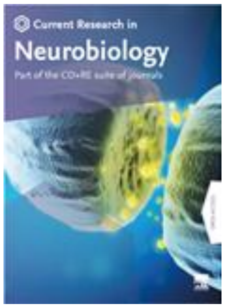


**Manuscript Title:** Protocol for a Wnt reporter assay to measure its activity in human neural stem cells derived from induced pluripotent stem cells


| | |
|---|---|
| Received | Mar 13, 2023 |
| 1st Decision | May 08, 2023 |
| 1st Revision Submitted | May 24, 2023 |
| Accepted | Jun 08, 2023 |


---

## 1st Decision letter

**Reference:** CRNEUR-D-23-00021
**Title:** Protocol for a Wnt reporter assay to measure its activity in human neural stem cells derived from induced pluripotent stem cells
**Journal:** Current Research in Neurobiology

Dear Prof. Dr. Grünblatt,

Thank you for submitting your manuscript to Current Research in Neurobiology.

I have completed my evaluation of your manuscript. The reviewers recommend reconsideration of your manuscript following minor revision and modification. I invite you to resubmit your manuscript after addressing the comments below. Please resubmit your revised manuscript by Jun 07, 2023.

When revising your manuscript, please consider all issues mentioned in the reviewers' comments carefully: please outline every change made in response to their comments and provide suitable rebuttals for any comments not addressed. Please note that your revised submission may need to be re-reviewed.

Current Research in Neurobiology values your contribution and I look forward to receiving your revised manuscript.

Kind regards,

Anna S Mitchell, Ph.D.
Editor in Chief
Current Research in Neurobiology

## Comments from Editors and Reviewers:

The expert reviewers and I agree that your manuscript is important and timely in the field of stem cell research. However, it requires some revisions to clarify the methods and data, and improve the readability of the figures. Please address all the reviewers comments (as detailed below) when you submit your revised manuscript in due course.

**Reviewer #1:**

The article describes an important novel tool to investigate WNT signalling in iPSC-derived neural precursor cells. The approach is going to be useful in both comparing WNT signalling in patient and healthy control subject derived cells, as well as in evaluating possible treatments and their effect, or lack thereof, on WNT signalling: The science behind the article is sound, and the results support the possibility to use the described reporter system in NPCs, and perhaps other cell types. Great work.

What lacks is the final editing effort.

For example, in Methods, some sections are written as do this then do this. Others are this was done, then that was done. The style of the protocol should be consistent throughout.

Or, all of a sudden (starting line 268) you start to talk about stable transfection, and why using it in iPSC is less feasible. The section should be re-written in a more logical and clear way, perhaps earlier in the article, explaining why you chose transient transfection over stable transfection, and why you used NPC rather than iPSC.

Abbreviations should be presented, and grouped somewhere in the abstract or immediately after. For example, you mention TCF/LEF responsive element already in the abstract, line 29, but only line 63 explains what it stands for. Please check all abbreviations are explained.

Supplementary figures and their legends also contain inconsistencies (or typos). For example, supplementary figure 1 describes the characterisation and quality control of iPSCs used in the study to produce NPCs. However, the figure legend says: (D) Immunofluorescence images from NSCs to show positive protein expression of SSEA etc. Did you mean iPSC? Please check through all figures and their legends. Or supplementary table 4 - title states NPCs, table headers say iPSC. Please double check.

Overall, consider asking a native speaker to read through main text the article to make the language more streamlined.

**Reviewer #2:**

This report describes a useful approach to measuring Wnt signalling activity in human iPSC-derived neuronal cells. It is therfore of value for the research community.

Comments

1. The iPSC are derived from two male individuals. I doubt different results would have been obtained if the origin of the cells were female, however I had to look into the supplemental information to find the

cell origins. It would be helpful for the reader if the sex of the cells was made clear in the main text

2. Table 1 shows considerable variation of signal from one close to the next (very different EC50s) Although the results are generally internally consistent for the different treatments, it is not clear why such large variation should be present. Do the authors believe that this is due to clonal variation in Wnt3a response (ie. some lines being more Wnt3a sensitive), or other causes?

3. Figure 2 shows the aggregate response across the 4 cell lines, but given the differential Wnt3a response of each line, can this be justified ? The figure legend needs to provide more information about what is meant by % Wnt activity. Also there is not statistical measure. For a regression analysis should the authors show the specitic data points, the calculated regression curve and the r value, to should the likelihood that the datsa fits the curve
Minor typos.

line 56 "and alas c)" - I'm not sure what this means

line 102: "retreived from Promeg" should this be either purchased or provided as a a gift.

line 244: "the plasmid was integer" - this does not make sense

**Reviewer #3:**

The present article is highlighting an easier way to screen for Wnt signaling in neural stem cell. The design of experiments were well performed, comparing transfection efficiency from single or double transfected line. Four lines were compared during the process, scanning for differences in transfection efficiency. The authors have also combined the cell line to quantify the overall response to the Wnt activation using these plasmid.
In general, this paper brings a reliable protocol to the field of Wnt signaling investigation and cover a part where complex protocols involving viruses production and random insertion into the genome was used to investigate such a pathway.
From a stem cell point a view, the paper is missing some data on how strong is the protocol when it comes to other types of neural stem cells (differentiated through other protocols). Only the supplementary material specifies what type of neural stem cells is used in the paper. The readers need more specificity as the transfection efficiency can differ from neural cell type origin (i.e. because of cell cycling differences, cell size, type of neural stem cells, maturation...). Please discuss this point in the paper.

Other questions:
L140 does the accutase remain with the cells or centrifugation steps
L153 To transfect 26 or 96 wells ?
L165 does the authors compensate for the HSA in other wells ?
L244 do the author checked for random plasmid integration ?

# 1st Author Response Letter

## Response to comments from Editors and Reviewers:

Dear Dr. Mitchell, Dear Reviewers

We would like to thank the editor and the reviewers for revising the manuscript. We have addressed all of the reviewers' comments and made the necessary changes. Please find our responses to the reviewers' comments below, answered point by point.

**Comments from Reviewer 1**:

The article describes an important novel tool to investigate WNT signalling in iPSC-derived neural precursor cells. The approach is going to be useful in both comparing WNT signalling in patient and healthy control subject derived cells, as well as in evaluating possible treatments and their effect, or lack thereof, on WNT signalling: The science behind the article is sound, and the results support the possibility to use the described reporter system in NPCs, and perhaps other cell types. Great work.

**Reply:** We would like to thank the reviewer for analyzing our manuscript and for the positive opinion about it.

1. What lacks is the final editing effort.

For example, in Methods, some sections are written as do this then do this. Others are this was done, then that was done. The style of the protocol should be consistent throughout.

**Reply:** We appreciate the reviewer's suggestion and therefore, we have updated the methods section for a consistent writing style.

2. Or, all of a sudden (starting line 268) you start to talk about stable transfection, and why using it in iPSC is less feasible. The section should be re-written in a more logical and clear way, perhaps earlier in the article, explaining why you chose transient transfection over stable transfection, and why you used NPC rather than iPSC.

**Reply:** We apologize for the lack of clarity under the Discussion. We have rewritten the section in a chorological order to justify the choice of transient transfection in NSCs in a more logical manner (starting from line 250).

3. Abbreviations should be presented, and grouped somewhere in the abstract or immediately after. For example, you mention TCF/LEF responsive element already in the abstract, line 29, but only line 63 explains what it stands for. Please check all abbreviations are explained.

**Reply:** We thank the reviewer for the attention to this detail. We have added what TCF/LEF stands for in the abstract and rechecked the other abbreviations throughout the manuscript. We added also an abbreviation table to help the reader throughout the manuscript.

4. Supplementary figures and their legends also contain inconsistencies (or typos). For example, supplementary figure 1 describes the characterisation and quality control of iPSCs used in the study to

produce NPCs. However, the figure legend says: (D) Immunofluorescence images from NSCs to show positive protein expression of SSEA etc. Did you mean iPSC? Please check through all figures and their legends. Or supplementary table 4 - title states NPCs, table headers say iPSC. Please double check.

**Reply:** We thank the reviewer for pointing out these typos on Supplementary Table 4. They have been properly corrected. Since the submission of this paper, we have now published papers describing the generation and characterization of both K013's and K015's iPSCs, therefore we have deleted Supplementary Figure 1 and properly cited these papers in the manuscript. Initially, this figure had been purposefully added to the manuscript to show the pluripotency state of the iPSCs, from which the NSCs were generated.

5. Overall, consider asking a native speaker to read through main text the article to make the language more streamlined.

**Reply:** We would like to thank the reviewer for the suggestion and confirm that an English native speaker has reviewed the manuscript.

**Comments from Reviewer 2:**

This report describes a useful approach to measuring Wnt signalling activity in human iPSCderived neuronal cells. It is therfore of value for the research community.

**Reply:** We thank the reviewer for the positive feedback to our methodological manuscript.

1. The iPSC are derived from two male individuals. I doubt different results would have been obtained if the origin of the cells were female, however I had to look into the supplemental information to find the cell origins. It would be helpful for the reader if the sex of the cells was made clear in the main text

**Reply:** We apologize for the lack of clarity about the gender of our tested cell lines. The gender of individuals from which the NSCs were derived is now available on line 123.

2. Table 1 shows considerable variation of signal from one close to the next (very different EC50s) Although the results are generally internally consistent for the different treatments, it is not clear why such large variation should be present. Do the authors believe that this is due to clonal variation in Wnt3a response (ie. some lines being more Wnt3a sensitive), or other causes?

**Reply:** We believe that one of the main causes for this variation could be the so-called "intraindividual variability" in iPSC clones derived from the same donor, which has been previously described (1, 2). As reported in the literature, this variability could be introduced by epigenetic differences or even de novo mutations derived from cell culture (3), leading to possible differential Wnt signaling response. Our internal CNV analysis (data not shown) indeed show that different clones from the same donor might present slight differential CNVs. Nevertheless, these genetic variations still do not compromise our Wnt signaling investigation.

3. Figure 2 shows the aggregate response across the 4 cell lines, but given the differential Wnt3a response of each line, can this be justified ? The figure legend needs to provide more information about what is meant by % Wnt activity. Also there is not statistical measure. For a regression analysis should the authors show the specitic data points, the calculated regression curve and the r value, to should the likelihood that the datsa fits the curve

**Reply:** We appreciate the reviewer's concern and for pointing out the details about statistical analysis. The highest Wnt activity (100%) for the agonists was set as the maximum mean of individual percentages obtained when the four cell lines are plotted together. As for DKK1, 26% of Wnt activity was determined as the mean of the individual percentages from wells treated with DKK1's vehicle (containing only Wnt3a's EC26) (lines 229-233). We have combined the cell lines with the purpose to representatively show that a non-linear regression across a dose-response can be constructed and therefore, the EC50 could be calculated from a group of cell lines of interest. The legend of figure 2 was adjusted and statistical details were added to Supplementary Table 6.

4. Minor typos.

line 56 "and alas c)" - I'm not sure what this means

line 102: "retreived from Promeg" should this be either purchased or provided as a a gift.

line 244: "the plasmid was integer" - this does not make sense

**Reply:** We would like to thank the reviewer for noticing those points. The term "alas" was removed for better understanding (line 59), while "retrieved" was replaced by "provided as a gift" (line 105) and the sentence "the plasmid was integer" was rephrased to "the integrity of the plasmid was preserved" (line 290).

**Comments from Reviewer 3:**

The present article is highlighting an easier way to screen for Wnt signaling in neural stem cell. The design of experiments were well performed, comparing transfection efficiency from single or double transfected line. Four lines were compared during the process, scanning for differences in transfection efficiency. The authors have also combined the cell line to quantify the overall response to the Wnt activation using these plasmid. In general, this paper brings a reliable protocol to the field of Wnt signaling investigation and cover a part where complex protocols involving viruses production and random insertion into the genome was used to investigate such a pathway. From a stem cell point a view, the paper is missing some data on how strong is the protocol when it comes to other types of neural stem cells (differentiated through other protocols). Only the supplementary material specifies what type of neural stem cells is used in the paper. The readers need more specificity as the transfection efficiency can differ from neural cell type origin (i.e. because of cell cycling differences, cell size, type of neural stem cells, maturation...). Please discuss this point in the paper.

**Reply:** We would like to thank the reviewer for their opinion. We have added a paragraph where we discuss more about the influence of those factors in transfection efficiency (lines 296-305).

1. L140 does the accutase remain with the cells or centrifugation steps

**Reply:** We thank the reviewer for noticing this important step. Accutase is removed prior to the cell counting by centrifugation at 300 xg for 4 minutes (line 145).

2. L153 To transfect 26 or 96 wells ?

**Reply:** We apologize for the unclear statement. The transfection protocol described on section 2.4 of the methods section is meant for 26 wells of a 96-well plate, to be adjusted proportionally depending on the total amount of wells one would have to transfect. The paragraph was rephrased on lines 162-166.

3. L165 does the authors compensate for the HSA in other wells ?

**Reply:** We appreciate the reviewer's concern about the concentration of HSA in the wells tested for Wnt3a. Each 96-well treated with the highest Wnt3a concentration (800 ng/mL) receives 3.2 µL of Wnt3a + 0.1% HSA in PBS diluted in 36.8 µL of Neural Expansion Media, being this volume the highest possible amount of HSA that our NSCs are treated with. In terms of concentration, this means that these wells end up with the negligible concentration of only 0.008% HSA each. This is the reason why this compound was not normalized across the different Wnt3a concentrations.

4. L244 do the author checked for random plasmid integration ?

**Reply:** We have not checked for plasmid integration. It is possible that the confounding term on line 244 was "integer". "Integer" was initially meant to describe the integrity of the plasmid after bacterial transformation, and was not really related to integration. We have rephrased the sentence to a clearer version, which can now be found on line 290-291.

Thank you very much for considering our manuscript and providing constructive comments to its improvement. We appreciate your time and look forward to your response.

References

1. Matsa E, Burridge PW, Yu KH, Ahrens JH, Termglinchan V, Wu H, et al. Transcriptome Profiling of Patient-Specific Human iPSC-Cardiomyocytes Predicts Individual Drug Safety and Efficacy Responses In Vitro. Cell Stem Cell. 2016;19(3):311-25.

2. Elanzew A, Niessing B, Langendoerfer D, Rippel O, Piotrowski T, Schenk F, et al. The StemCellFactory: A Modular System Integration for Automated Generation and Expansion of Human Induced Pluripotent Stem Cells. Front Bioeng Biotechnol. 2020;8:580352.

3. Beekhuis-Hoekstra SD, Watanabe K, Werme J, de Leeuw CA, Paliukhovich I, Li KW, et al. Systematic assessment of variability in the proteome of iPSC derivatives. Stem Cell Res. 2021;56:102512.

## Accept Letter

Dear Prof. Dr. Grünblatt,

Thank you for submitting your manuscript to Current Research in Neurobiology.

I am pleased to inform you that your manuscript has been accepted for publication.  Congratulations.

My comments, and any reviewer comments, are below.

Your accepted manuscript will now be transferred to our production department. We will create a proof which you will be asked to check, and you will also be asked to complete a number of online forms required for publication. If we need additional information from you during the production process, we will contact you directly.

We appreciate and value your contribution to Current Research in Neurobiology. We regularly invite authors of recently published manuscript to participate in the peer review process. If you were not

already part of the journal's reviewer pool, you have now been added to it. We look forward to your continued participation in our journal, and we hope you will consider us again for future submissions.

*CRNEUR* aims to be a unique, community-led journal, as highlighted in the Editorial Introduction. As part of this vision, we will be regularly seeking input from the scientific community and encourage you and your co-authors to take the survey.

We would also like to invite you to take part in our CRNEUR Author Question & Answer (Q&A), which could get published alongside your article and help to promote it. We suspect you might have an interesting story of perseverance or team work that was required for the research study to complete, or a diversity of perspectives that you might share, as a way of inspiring others about neuroscience.

Kind regards,

Anna S Mitchell, Ph.D.
Editor in Chief
Current Research in Neurobiology

Editor and Reviewer comments:

Reviewer 1: All concerns have been properly addressed, no further comments. Great work.

Reviewer 2: I am happy with the revsions

-------- *End of Review Comments* --------
